# German Environmental Survey for Children and Adolescents 2014-2017 (GerES V) – the environmental module of KiGGS Wave 2

**DOI:** 10.17886/RKI-GBE-2017-108

**Published:** 2017-09-27

**Authors:** Christine Schulz, Marike Kolossa-Gehring, Andreas Gies, for the GerES study group

**Affiliations:** German Environment Agency, Department of Environmental Hygiene, Berlin

**Keywords:** GERES, HUMAN BIOMONITORING, MONITORING OF NEIGHBOURHOODS AND LIVING ENVIRONMENTS, HEALTH MONITORING

## Abstract

Health-relevant exposures to environmental pollutants, fungi, bacteria, noise, and air pollution have to be identified at an early stage. At the same time, impacts on health and their potential environmental causes need to be investigated and documented. The German Environmental Survey for Children and Adolescents 2014-2017 (GerES V) is the environmental module of KiGGS Wave 2 of the Robert Koch Institute and takes a deeper look at the sections living conditions and health status of the KiGGS study. GerES V collects up-to-date information on the exposure of children and adolescents in Germany aged 3 to 17 to chemicals and investigates chemical and physical environmental pollutants in their living environments. The survey contributes to identifying environmental hazards and measures that effectively reduce or prevent such hazards in order to protect and promote the health of the young generation.

## 1. Background and objective

The German Environmental Surveys are large-scale cross-sectional studies. They strive to collect representative data on the German population’s health-related and domestic exposure to environmental exposures. This data is constantly updated and evaluated to provide science-based advice to policy-makers and the public. Data and analyses are made available to the scientific community and the general population. They constitute key elements in the health-related federal monitoring of the environment (GUB) and national-level environmental reporting. They provide a basis for evaluating the population’s exposure to health-relevant environmental pollutants and deriving basic data for exposure analyses and risk assessments [1].

The reference values for blood and urine levels of environmental pollutants are one example of how these measurement results are used. They are derived by the German Human Biomonitoring Commission of the German Environment Agency. These reference values indicate whether a person’s exposure to pollutants is above average.

GerES data can reveal changes over time and regional differences in exposure. This allows the identification of regions and population groups that are particularly vulnerable (hot spots and at risk groups). Identifying the sources of pollutants and exposure pathways and analysing associations between environmental pollutants and diseases helps to develop strategies to minimise exposure and develop targeted environmental policy measures.

**GerES V**

Fifth cycle of the German Environmental Survey 2014-2017

**Acronym: GerES** - **Ger**man **E**nvironmental **S**urvey

**Implementation:** German Environment Agency

**Aim:** Providing reliable information on the exposure of children and adolescents aged 3 to 17 in Germany to chemicals as well as on the exposure to chemical and physical environmental stressors of young people in their living environments.

**Study design** Cross-sectional examination and interview survey

**Population:** Children and adolescents with permanent residence in Germany

**Sampling:** GerES V participants were randomly selected from the cross-sectional sample of KiGGS Wave 2 (registry office sample). An invitation to GerES V required prior participation in KiGGS Wave 2.

**Age range:** 3-17 years

**Sample size:** approximately 2,500 participants

**Survey period:** January 2015-June 2017

More information is available at www.uba.de/geres

The data collected by GerES also provide a basis for formulating German positions on the scientific development of the European chemicals regulation REACH (registration, evaluation, authorisation and restriction of chemicals).

The German Environment Agency (UBA) has been implementing GerES cycles since 1985 in close cooperation with the health surveys of the Robert Koch Institute (RKI) [2], which is why the German Environmental Survey recently supplemented the KiGGS baseline study [3].

Table 1 shows GerES samples and response rates and the surveys that each cycle cooperated with.

KiGGS Wave 2 collected both socio-demographic data and information on health status and behaviour, living conditions, protective and risk factors, as well as on preventive healthcare. Their combination with the data collected by GerES V on the exposure of children and adolescents in Germany to harmful substances allows researchers to establish new hypotheses regarding environmental health impacts. A particular analytical focus is on types of environmental hazards and their correlation with health indicators, but also on the German population’s environment-related disease burden and on the analysis and evaluation of economic and social correlations.

## 2. Methodology

### 2.1 Study design and sampling

The sample of the German Environmental Survey for Children and Adolescents 2014-2017, GerES V, consists of the participants aged 3 to 17 who were examined as part of the cross-sectional sample of KiGGS Wave 2. KiGGS target population and sampling is discussed in detail in the article New data for action. Data collection for KiGGS Wave 2 has been completed in this edition of the Journal of Health Monitoring. Children and adolescents were randomly drawn from the gross sample and assigned to the GerES V study during sampling for KiGGS Wave 2 and independently of their previous participation in KiGGS. An invitation to GerES V was bound to prior participation in KiGGS Wave 2.

**Figure 1:**
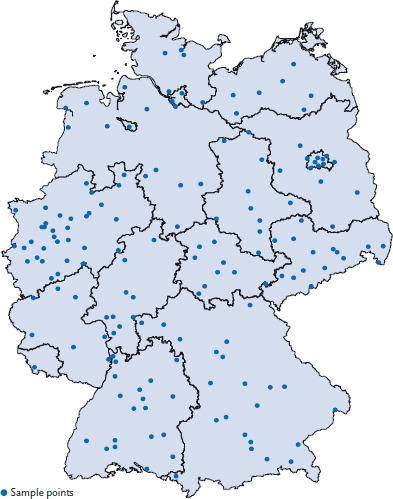
Sample points of GerES V Source: RKI

All GerES V participants were selected from the pool of KiGGS Wave 2 participants. At the beginning of their visit to the examination centre, they were informed about the goals and content of the survey and motivated to participate. RKI staff in particular drew on the GerES V survey flyer and invitation letter. KiGGS Wave 2 participants who showed interest in GerES V were asked to sign consent forms for their data to be shared, to allow GerES staff to contact them, and if possible also for a blood sample to be forwarded to the UBA. Families interested in participating then received a letter with an appointment for the examination and interview at their home from Kantar Health GmbH, the company commissioned by the UBA to conduct the field work for GerES V. Participants were expected to confirm the appointment in writing, which then took place about ten days later. Figure 1 shows the sample points.

The study strictly complies with data protection regulations and has been approved by Germany’s Federal Commissioner for Data Protection and Freedom of Information. Participation in the survey was voluntary. Participants in the survey or their legal guardians were informed about the goals and content of the survey as well as about data privacy, and provided their informed consent. The study received a positive vote from the ethics commission of the Berlin Chamber of Physicians (Ärztekammer Berlin, Eth-14/14).

### 2.2 Assessment methods and testing instruments

Scientific validity was the key criterion behind the selection of survey methods and instruments. Additional aspects that were also considered were: practicality of instruments in the field, the amount of time participants would have to invest, and the feasibility of ensuring contamination-free sampling and the contamination-free storage and transport of samples. The UBA successfully tested the survey methods and instruments in a GerES V pilot study [7].

**KiGGS Wave 2**

Second follow-up to the German Health Interview and Examination Survey for Children and Adolescents

**Data owner:** Robert Koch Institute

**Aim:** Providing reliable information on health status, health-related behaviour, living conditions, protective and risk factors, and health care among children, adolescents and young adults living in Germany, with the possibility of trend and longitudinal analyses.

**Study design**

Combined cross-sectional and cohort study conducted as an examination and interview survey

**KiGGS cross-sectional study**

**Population:** Children and adolescents with permanent residence in Germany

**Sampling:** Samples from official residency registries - randomly selected children and adolescents from the 167 cities and municipalities covered by the KiGGS baseline study

**Age range:** 0-17 years

**Sample size:** Approximately 15,000 participants

**KiGGS cohort study**

**Sampling:** Re-invitation of everyone who took part in the KiGGS baseline study (2003-2006; aged between 0 and 17 at that time) and who was willing to participate in a follow-up

**Age range:** 10-29 years

**Sample size:** Approximately 10,000 follow-up participants

**Survey period:** September 2014-August 2017

**Modules:** BELLA, EsKiMo, GerES, KiESEL, MoMo

More information is available at www.kiggs-studie.de/english

GerES V data collection consisted of a standardised interview, human biomonitoring (blood and morning urine samples), indoor monitoring (house dust, indoor air and drinking water samples), noise level measurements and – for the first time in GerES V – a measurement of the indoor concentration of ultrafine particles and a measurement of the particulate matter (PM2.5) concentrations indoors and outdoors. Blood samples were taken only once after participants signed the consent form in KiGGS Wave 2.

CAPI (Computer Assisted Personal Interview) was used to collect data on the neighbourhood, flat use, equipment and furniture, use of products, amount of time spent indoors and in which rooms, pets, passive and active smoking, dental status, clothing, dietary habits, subjectively perceived levels of noise and to document the samples and measures taken. Data on potentially environment-related health hazards faced by children and adolescents were recorded in a self-administered questionnaire. A further self-administered questionnaire allowed respondents to provide feedback on the process and content of the individual examination programmes (satisfaction surveys).

The samples are tested for environmental pollutants that are known or believed to cause health impacts and may lead to exposure of the general population. Quality assured analysis methods for these substances have to be available. Tests were conducted for example for phthalates and substitute plasticisers, cosmetics ingredients such as UV filters and preservatives, heavy metals such as lead and quicksilver, volatile organic compounds such as benzene, polycyclic aromatic hydrocarbons (PAH), per- and polyfluorinated compounds (PFAS) or polychlorinated biphenyls (PCB).

On the appointment day, participants were visited in their homes by a specially trained interviewer. The examinations and interviews took on average 90 minutes to complete and comprised the following components:

► Drinking water sample from the tap the family usually uses for drawing drinking water [8]
► A morning urine sample of participating children and adolescents
► A measurement of noise levels in front of the window of the room in which the child or adolescent sleeps
► A measurement of ultrafine particles in the room air of the child’s room
► A personal interview with the parents or guardians and with those children/adolescents aged 11 and older
► A self-administered “Questionnaire on diseases and health problems suffered by the child”
► The self-administered “Satisfaction survey” questionnaire.

Moreover, in some randomly selected households:

► Additional drinking water samples
► A full vacuum cleaner bag
► Volatile organic environmental pollutants in the room air collected with passive samplers on seven subsequent days
► Suspended particulate matter collectors for active collection of indoor and outdoor particulate matter on seven subsequent days

The GerES V field phase began on 15 January 2015 and ended on 21 June 2017.

## 3. Discussion and outlook

GerES V provides the basis to analyse a broad range of politically and scientifically relevant questions. Some examples include:

1. To which degree is the population exposed to chemical compounds for which no monitoring method previously existed? A collaboration project between the Federal Ministry for the Environment, Nature Conservation, Building and Nuclear Safety (BMUB) and the German chemical industry association (VCI) launched in 2010 has been developing up to five new methods per year to measure pollutants accumulated in the body. These methods are to be pioneered in GerES. The goal is to develop up to fifty new methods over the coming years [9].
2. Which impact do room setup and use have on indoor pollution levels with carcinogenic polycyclic aromatic hydrocarbons? The increasing popularity of wooden fire stoves potentially increases risks, which is why analysis here focuses on particulate matter that PAH potentially adsorbs to.
3. What are the actual contamination levels of drinking water? Are the parameters of the household drinking water regulation for lead, nickel and copper being met?
4. Do social status or gender impact a person’s exposure to pollutants? In terms of environmental justice, analysis here is aimed at identifying the social factors that influence exposure to pollutants, indoor pollutants and stressors in the living environment such as noise levels.
5. How great is the loss of healthy life years and quality of life due to the population’s exposure to harmful environmental impacts? Which environmental impacts on average cause greater disease burdens than others?
6. Which healthcare costs do environment-related diseases cause?

Initial results should become available around one and a half years after conclusion of the field phase. The respondents’ high willingness to participate in the survey and the high share of correctly collected samples and measurements have created the basis for a successful conclusion of GerES V. The results will contribute to making the environment in Germany even more liveable and healthy for children and adolescents and their families. With the German environmental survey on health, the RKI and UBA together make an important contribution to overall efforts in protecting health and the environment.

## Key statements

- For GerES V drinking water, urine, blood, dust and air samples are analyzed for environmental pollutants.
- Particulate matter contamination and noise are measured in neighbourhoods and analysed in combination with questionnaire responses.
- The results provide a basis for decisions on environmental health regulations and laws.
- The results help raise awareness in Germany of the young generation’s exposure to environmental pollutants.

## Figures and Tables

**Table 1:** GerES survey waves Own table

	GerES I	GerES II a+b	GerES III	GerES IV	GerES V
Regional dimension	West Germany	a) West Germany b) East Germany	Germany	Germany	Germany
Study period	1985-1986	a) 1990-1991 b) 1991-1992	1997-1999	2003-2006	2014-2017
Age range, in years	25-69	a) 25-69 & 6-14 b) 18-79 & 6-17	18-69	3-14	3-17
Net sample (N)	2,731	a) 2,524 and 453 b) 1,763 and 359	4,822	1,790	Objective: 2,500
Response rate (%)	73.0	a) 63.1 b) 69.0	54.5	77.3	To be calculated after study concludes
Co-operation	National examination survey t_0_ 1984-1986 [4]	National examination survey t_2_ 1990-1992 [4]	Federal health survey 1998 [5]	KiGGS baseline study [6]	KiGGS Wave 2
